# Deep Eutectic Solvents for Sustainable Extraction of Bioactive Compounds from Biomass: Mechanistic Insights and Scale-Up Challenges

**DOI:** 10.3390/molecules31050880

**Published:** 2026-03-06

**Authors:** Selin Şahin, Ebru Kurtulbaş, İrem Toprakçı, Farooq Anwar, Rahim Khan, Zeynep Ciğeroğlu, Atike İnce Yardımcı, Mehmet Torun, Ferhan Balcı Torun, Seid Reza Falsafi

**Affiliations:** 1 Chemical Engineering Department, Faculty of Engineering, Istanbul University-Cerrahpasa, Avcilar, 34320 Istanbul, Turkey; 2 Faculty of Food Science and Technology, University Putra Malaysia, Serdang 43400, Selangor, Malaysia; 3 Institute of Chemistry, University of Sargodha, Sargodha 40100, Pakistan; 4 Faculty of Health Sciences, Shinawatra University, Bang Toey, Pathum Thani 12160, Thailand; 5 Chemical Engineering Department, Faculty of Engineering and Natural Sciences, Uşak University, 64300 Uşak, Turkey; 6 Technology Transfer Office, Uşak University, 64300 Uşak, Turkey; 7 Food Engineering Department, Faculty of Engineering, Akdeniz University, 07058 Antalya, Turkey; 8 Department of Gastronomy and Culinary Art, Faculty of Tourism, Akdeniz University, 07058 Antalya, Turkey; 9 Food Science and Technology Division, Agricultural Engineering Research Department, Safiabad Agricultural and Natural Resources Research and Education Center, AREEO, Dezful P.O. Box 333, Iran

**Keywords:** sustainable chemistry, green solvents, process intensification, natural product chemistry, mass transfer

## Abstract

Deep eutectic solvents (DESs) have emerged as promising green alternatives to conventional organic solvents for the extraction of bioactive compounds from natural matrices because of their tunable physicochemical properties, low toxicity, and environmental compatibility. However, most existing reviews primarily focus on application-based results, with limited mechanistic and process engineering interpretations necessary for industrial applications. This review provides a comprehensive analysis of DES-based extraction from the perspective of separation and process engineering, emphasizing the relationships between DES composition, physicochemical properties, mass-transfer behavior, and extraction performance. Key parameters, including viscosity, hydrogen bonding interactions, solvent-to-feed ratio, temperature, and water content, are critically evaluated in terms of their influence on extraction efficiency, selectivity, and scalability. Furthermore, solvent recovery, process intensification strategies, and industrial implementation challenges are discussed to bridge the gap between laboratory research and large-scale application. By integrating mechanistic insights with process-level considerations, this review provides a systematic framework for the rational design and optimization of DES-based extraction processes as sustainable and scalable-separation technologies.

## 1. Introduction

Bioactive compounds are natural chemical substances that significantly affect human and animal health. They are obtained from living organisms (plants, animals, and microorganisms). Therefore, they are known to be generally regarded as safe (GRAS) [1]. However, their classification as GRAS is not universal, as it depends on the specific compound, its intended application, concentration and the relevant regulatory framework. They have many health benefits, such as anticancer, antioxidant, anti-inflammatory, antimicrobial, antiallergic and antidiabetic properties [2]. The significance of bioactive components has recently increased, driven by a growing interest in natural products and the increasing resistance of metabolic processes to synthetic drugs [3]. Bioactive components have also received attention as functional additives in foods and nutraceuticals. These components have positive effects on health by enhancing the functional properties of products to prevent or manage various diseases [4]. In addition, bioactive components have significant potential for the evaluation of food and agricultural industry waste [5]. Bioactive components play an important role in areas such as the protection and improvement of human and animal health, new drug discovery, development of functional foods and nutraceutical products, and evaluation of food and agricultural industry waste [6]. Therefore, they have been intensively researched in terms of discovery, isolation, characterization, and applications [7,8,9,10].

The extraction and separation of bioactive compounds from natural matrices play a central role in the food, pharmaceutical, cosmetic and biotechnology industries. These compounds (polyphenols, alkaloids, terpenoids and carotenoids) are typically embedded within complex biological structures, making their efficient recovery highly dependent on solvent properties and mass-transfer phenomena. The use of volatile organic solvents in conventional extraction methods raises issues of toxicity, energy consumption, environmental effects, and selectivity. These disadvantages have spurred the quest for substitute solvent systems that can increase extraction efficiency while permitting safer and more sustainable process operations from the standpoint of separation engineering [11,12]. These methods may also negatively affect the structural stability of the target bioactive compounds [13,14,15]. Consequently, the need for more environmentally friendly, effective, and economical methods has increased.

Innovative extraction techniques based on green chemistry principles have recently gained attention for overcoming these challenges [16,17,18]. These techniques focus on reducing the toxicity of solvents, increasing energy efficiency and ensuring the sustainability of the processes. In addition to solvent toxicity and environmental impact, the performance of extraction and concentration processes should also be evaluated based on solute recovery efficiency, preservation of target compounds, selectivity and scalability. High solute recovery must be achieved without compromising the chemical integrity or bioactivity of the extracted compounds, as excessive thermal or chemical stress may lead to degradation or structural modification. Selectivity is particularly critical when dealing with complex natural matrices, as it determines the enrichment of target bioactives relative to co-extracted impurities. Furthermore, process scalability, including solvent-to-feed ratio, energy demand, solvent recyclability and compatibility with intensified extraction techniques, plays a decisive role in translating laboratory-scale DES-based extractions into industrially viable processes.

Deep eutectic solvents (DESs) are a recent innovation [19]. DESs are characterized by their low toxicity, biodegradability, low cost, and malleability. Therefore, they are environmentally friendly alternatives for the extraction of bioactive compounds. Furthermore, some properties of DESs, such as polarity, can be adjusted by selecting appropriate hydrogen bond acceptors (HBAs) and donors (HBDs). However, the integration of DESs with advanced techniques such as ultrasound-assisted extraction (UAE) or microwave-assisted extraction (MAE) can significantly improve the extraction efficiency, reduce solvent consumption and shorten the extraction time. In contrast, the traditional extraction methods, such as Soxhlet, are time-consuming and require large volumes of solvents [20]. Ionic liquids (ILs) are also known as green solvents. However, ILs cannot be easily synthesized in contrast to DESs. DESs are prepared from inexpensive and readily available components, making them a more cost-effective and scalable option [21].

In conclusion, the DES application is a green chemistry perspective and a high-potential approach for the extraction of biological components. DES has a significant future due to its sustainability, environmental friendliness and flexibility considering the challenges associated with traditional organic solvents such as toxicity, limited selectivity, and high energy consumption [22]. These properties of DESs, along with improved extraction efficiency, make them attractive for various applications, including the extraction of bioactive compounds from natural sources.

The present examines DES-based extraction from a separation and process-engineering perspective. Rather than providing a purely application-driven overview, this work emphasizes the mechanistic links between DES composition, physicochemical properties, mass-transfer behavior and extraction performance. Important performance metrics such as solvent viscosity, diffusion limitations, solvent-to-solid ratio, energy input and solvent recyclability are systematically discussed. Furthermore, the integration of DESs with process-intensification techniques is assessed in terms of their ability to mitigate transport limitations and enhance separation performance. Last but not least, this review highlights the major challenges hindering industrial implementation of DES-based extraction. This study offers a systematic framework for the rational design and advancement of DES-based extraction procedures as scalable and sustainable separation technologies by identifying existing knowledge gaps and specifying future research goals.

## 2. Fundamentals and Properties of Deep Eutectic Solvents

### 2.1. Definition and Classification

**Figure 1:**
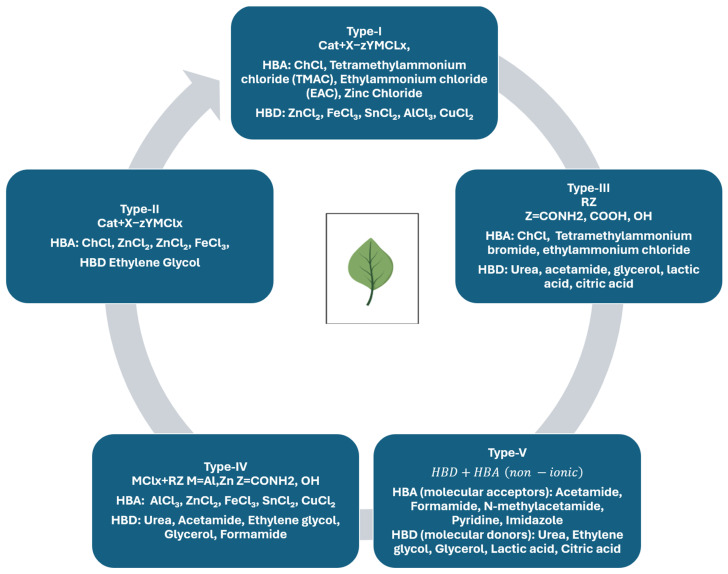
Classification of deep eutectic solvents that outlines their general formulas, along with examples of hydrogen bond acceptor and hydrogen bond donor components.

DESs are eutectic mixtures formed by strong HBA-HBD interactions, leading to a melting point far below that of the individual components. Because this hydrogen-bond network governs physicochemical properties and extraction performance, DESs are structurally classified into five types (Type I–V) (Figure 1). Cat+ usually consists of ammonium, sulfonium or phosphonium. X is a Lewis base, while Y is a Lewis or Brønsted acid. Finally, z is the number of Y molecules interacting with the corresponding anion [23]. Hydrogen bonding is dominant in Type V DESs consisting of nonionic molecular HBD and HBA [24]. Type III DESs are among the most widely studied DES types by researchers and are frequently used in applications. They usually contain non-toxic and biodegradable choline chloride (ChCl) as HBA. While the first Type III DESs were primarily based on ChCl, over time, many compounds were used in the formation of DESs. Accordingly, the Type I–V scheme is used as the primary structural classification throughout this review. Terms such as natural DESs (NADESs), hydrophobic DESs (HDESs), ternary DESs (TDESs) and supramolecular DESs (SUPRADESs) are treated as compositional/functional subclasses within this unified DES framework.

From a functional and application-oriented perspective, the terminology used to describe certain DES variants does not correspond to a structural classification; rather, these designations reflect their compositional origin, physicochemical characteristics, or intended application domains. HBAs mainly contain quaternary ammonium/phosphonium salts, while the most widely used HBDs are amides, carboxylic acids and alcohols. In addition, compounds such as sugars/sugar alcohols and amino acids have been used in the preparation of NADESs [25]. Additionally, there are also therapeutic DESs containing drug components such as lidocaine, ibuprofen and phenylacetic acid [26,27]. Although NADESs are sometimes considered Type III DESs, this is not entirely true, and NADESs are commonly categorized based on the nature of their primary components (e.g., sugar-based, organic acid-based, amino acid-based, or combinations thereof), rather than according to the structural Type I–V DES classification [28]. NADES formulations may include ionic components such as choline chloride; however, this does not constitute a distinct structural DES type. Many NADES systems consist exclusively of neutral components, such as sugars and other polyalcohols. Depending on their composition, NADESs may also comprise organic acids, amino acids, bases, or combinations of these natural metabolites, which collectively establish the hydrogen-bonding network responsible for eutectic formation [28].

Representative examples of structural DES Types (I–V) and compositional/functional variants are summarized in Table 1.

### 2.2. Physical and Chemical Properties

#### 2.2.1. Physical Properties

One of the defining characteristics of DESs is their low melting point, typically below room temperature, which allows them to remain liquid under standard conditions. The melting point of DESs can be adjusted by varying the composition of the HBD and HBA, providing flexibility for different applications. Density is another basic physical property of DESs. DESs tend to have a higher density than water and other common solvents, which is attributed to the strong intermolecular forces present in these mixtures. Most DESs show density values ranging from 1.0 to 1.3 g·cm^−3^ at 25 °C. However, the densities of DESs can be influenced by the nature and concentration, and the density of DESs based on metal salts is observed in the range of 1.3–1.6 g·cm^−3^ [45]. In contrast, HDESs can exhibit densities lower than 1 g·cm^−3^. The density of DES varies depending on temperature. It decreases linearly with increasing temperature [46]. In addition to temperature, density also depends on the HBD [47] and the molar ratios [48]. Higher density may influence phase separation behavior and solvent handling during downstream recovery steps, especially in liquid–liquid extraction or centrifugation-based separations.

Most of the DESs reported in the literature have very high viscosity values, above > 100 cP at room temperature. These high viscosity values are due to the strong hydrogen bonding between the components of DESs. However, viscosity can vary depending on the components and their ratio. Some DESs require relatively low viscosity for successful use in industrial applications. In the literature, Zhang et al. reported a very low viscosity value of 37 cP for ChCl:ethylene glycol (1:2) at 25 °C, while the viscosity values obtained for sugar-based DESs are 12.730 cP for ChCl:sorbitol (1:1) at 30 °C and 34.400 cP for ChCl:glucose at (1:1) 50 °C [49]. Gajardo-Parra et al. measured density and viscosity for 17 DESs based on ChCl+glycol between 293.15 and 333.15 K temperature for the mole ratios of HBA:HBD between 1:2 and 1:6 at 101.3 kPa [50]. The viscosity and density decreased with increasing temperature and glycol (HBD) ratio. It was determined that HBDs with longer carbon chains in DESs caused a decrease in density and an increase in viscosity at a constant temperature.

The elevated viscosity is one of the most characteristic physicochemical limitations of many DESs and can strongly influence their extraction performance. Owing to their extensive hydrogen-bonding network, DESs often present higher viscosity than conventional organic solvents, which reduces solvent diffusivity and limits penetration into the porous structure of natural matrices. This restriction in mass transfer may slow the release of intracellular bioactive compounds, such as phenolic acids and flavonoids, thereby decreasing extraction kinetics and recovery yields. In plant-based materials, efficient solute recovery depends on the ability of the solvent to permeate the cell wall and promote rapid solubilization of target compounds. Therefore, the high viscosity of DESs can represent a diffusion barrier, especially when extracting compounds trapped within complex lignocellulosic structures. To mitigate this drawback, viscosity-reduction strategies, such as moderate heating, dilution with controlled amounts of water, or the application of assisted extraction techniques, are commonly employed to enhance mass transfer and improve extraction efficiency. Thus, although DESs exhibit strong solvation capacity and high affinity toward bioactive compounds, their viscosity must be carefully optimized to maximize recovery from natural samples [51].

Many DESs, especially those containing salts, exhibit ionic conductivity. This makes them useful for electrochemistry and energy storage applications [52]. Viscosity directly affects conductivity. Therefore, most DESs tend to have poor ionic conductivity below 2 mS.cm^−1^ at room temperature. The viscosity value decreases, while the conductivity increases with increasing temperature [53]. The parameters affecting the conductivity of DESs are the molar ratio of the HBA/HBD, the characteristics of both the organic salt and the HBD, and the addition of water [54].

DESs generally exhibit good thermal stability (up to 200–300 °C), depending on the components utilized [55]. Gautam et al. studied the thermal conductivities of three DESs at temperatures between 298 and 343 K [56]. As the temperature increased, the thermal conductivity decreased slightly, showing a linear dependence across the investigated temperature range. DES-I and II indicated higher thermal conductivity than some ionic liquids [57], while DES-III exhibited a lower thermal conductivity because of sulfur and alkyl chain presence in HBD. These chains in the HBD caused a reduction in the density of the hydrogen bond, causing lower thermal conductivity.

The specific components of DESs largely determine their thermal stability. Delgado-Mellado et al. examined the thermal stability of various ChCl-based DESs formed using ethylene glycol, glycerol, phenylpropionic acid, malonic acid, phenylacetic acid, levulinic acid, glucose and urea as HBDs [55]. The results showed that the maximum operating temperatures determined by isothermal TGA were significantly lower than the initial decomposition temperatures of each DES examined. Thermal stability determines whether DESs can be reused after heating-assisted extraction processes, which is critical for sustainable, large-scale applications.

These physical properties, along with the ability to modify the composition of DESs, make them useful in a wide range of applications such as biocatalysis, electrochemical cells, solvent extraction and as green alternatives to traditional organic solvents.

#### 2.2.2. Chemical Properties

One of the key chemical properties of DESs is the extensive hydrogen bonds between HBD and HBA components. This hydrogen bonding significantly lowers the melting point of the mixture and provides the formation of a liquid phase at room temperature. In addition, the hydrogen bonds formed in DESs affect their ability to dissolve various substances. DESs can dissolve a wide variety of compounds, including ionic salts, transition metals and organic molecules by breaking chemical bonds or solvating ions [58]. This is particularly advantageous for extracting heat-sensitive bioactives, since liquefaction at mild conditions reduces thermal degradation and energy demand.

Guatam et al. synthesized ChCl-formic acid and ChCl-acetic acid DESs [59]. They indicated that the chloride ion formed a strong hydrogen bond with formic and acetic acids. Furthermore, the choline ion (HBD) facilitated the strong hydrogen bond formation. When the HBD is an organic/inorganic acid, alkali or amine, the resulting DESs will show mild acidity and alkalinity, which can be adjusted by altering the HBA:HBD molar ratio [29]. The chemical reactivity of these components can significantly influence the extraction of natural products. Acidic DESs based on carboxylic acids (e.g., formic, lactic, or acetic acid) may enhance the extraction efficiency of phenolic compounds, flavonoids, and alkaloids by promoting protonation or disrupting plant cell wall structures. In some cases, such acidic environments can stabilize certain compound classes, preventing oxidative degradation during extraction. However, strong acidity may also trigger undesired side reactions, such as esterification or hydrolysis of sensitive metabolites, potentially affecting compound integrity and selectivity. In particular, DESs based on ChCl are susceptible to thermal degradation when exposed to microwave treatment. Esterification can occur in certain acidic DESs where ChCl is mixed with carboxylic acids for an extended period at room temperature. Although each plant material is specific and there exists a vast variety of structurally different tannins, generally, acid-based DESs have shown a superior extraction capacity for tannins [60]. Similarly, DESs containing alcohol-based HBDs may participate in transesterification reactions under specific conditions. This could modify lipid-derived natural products or influence the recovery of ester-containing bioactives. Basic DESs prepared using amines as HBDs may improve the solubilization of acidic compounds through deprotonation and increase the selectivity toward specific target molecules [61].

DESs are extremely versatile solvents that can dissolve a wide variety of substances, including both polar and non-polar compounds, due to their strong intermolecular interactions. Mokhtapour et al. studied the solubility of six drugs in water and five different DESs at various temperatures, utilizing Dynamic Light Scattering spectroscopy to investigate the interactions between DESs and drugs [62]. The results showed that the drug crystals were insoluble in water, but the drug crystals dissolved in the DESs.

DESs are generally chemically stable and resist chemical degradation. However, their stability can be affected by their components. Stable Ti_3_C_2_T_x_ layers in DESs as an anti-oxidative dispersion medium maintained chemical oxidation stability for up to 28 weeks. DESs can remain hydrated during this process and form DES-water clusters when water is present, preventing water molecules from participating in the oxidation process [63]. DESs containing highly reactive components may exhibit reactivity with substrates such as metals or organic compounds. For example, DESs based on ChCl may react with metals.

With their ionic conductivity and electrochemical properties, DESs are used as electrolyte solutions in electrochemical processes. The ability of DESs to dissolve salts and metal ions makes them useful for applications such as batteries, supercapacitors and electroplating. However, the redox stability of a DES can vary depending on its components. DESs containing ChCl as HBA may be electrochemically stable, while some DESs may decompose under certain voltages [64]. Conductivity is relevant in electrically assisted extraction techniques, where ionic mobility can affect process efficiency.

In conclusion, the chemical properties of DESs are determined by their components, HBA/HBD species, ionic character, and metal coordination. DESs are generally chemically stable and have low volatility. Additionally, their reactive properties are customized. Therefore, viscosity, polarity, and thermal stability are essential to maximize extraction efficiency while ensuring scalable solvent recovery.

## 3. Designing the Deep Eutectic Solvents for Bioactive Components Extraction

**Figure 2:**
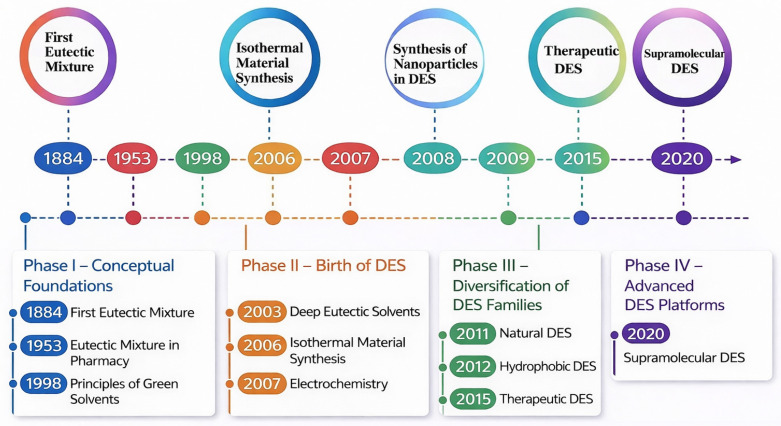
Conceptual evolution of deep eutectic solvents (DESs) and their compositional diversification into functional subclasses.

Deep eutectic solvents (DESs) are formed by combining suitable hydrogen bond acceptors (HBAs) and hydrogen bond donors (HBDs) at specific molar ratios, leading to significant melting point depression driven by strong hydrogen-bond interactions. Although terms such as NADES, TDES, HDES and SUPRADES are frequently used in the literature, they represent compositional or functional variations within a unified DES framework rather than distinct solvent families (Figure 2) [65]. Conventional DESs are HBA-HBD mixtures, NADES use natural components, TDES add a third component, HDES target non-polar compounds, and SUPRADES rely on molecular encapsulation, while all systems operate through the same hydrogen-bond-driven eutectic mechanism.

The efficiency of DES-based extraction mainly depends on solvent–solute affinity, HBA/HBD type and ratio, water content, temperature, and extraction time. These factors influence viscosity, polarity, and hydrogen bonding strength, thereby determining mass transfer and solubility. Water is commonly added to DESs to reduce viscosity and improve solvent penetration into plant matrices. Moderate water content enhances extraction efficiency, whereas excessive dilution weakens hydrogen-bond interactions and reduces selectivity. Similarly, adjusting the HBA/HBD ratio allows tuning of solvent polarity and viscosity for targeted extraction of specific bioactive compounds.

DES-based extraction is often performed under mild conditions, typically between 30 and 65 °C and within relatively short processing times. Ultrasound or microwave assistance further enhances mass transfer by facilitating cell disruption and the release of solute. Predictive tools such as COSMO-RS can also be applied to screen DES compositions and estimate solvent–solute interactions before experimental studies.

Strong hydrogen bonds formed by DESs are one of their main advantages since they improve the solubility of both polar and non-polar molecules equally. Incorporating water into DES formulations can reduce viscosity, which enhances solvent penetration into plant matrices and facilitates the extraction of bioactive components [66,67]. Studies have shown that varying the water content in DESs can lead to significant changes in extraction efficiency, with optimal water percentages enhancing the solvation of target compounds. Water addition to a DES reduces its viscosity, hence improving mass transfer and extraction efficiency [68]. The research by Florindo et al. showed water could lower DES viscosity by 10 to 200 times depending on the HBD utilized [69]. Ruesgas-Ramón et al. reported that the optimal values of the addition of water to DESs in extraction were in the range of 20–30% [70]. This is especially pertinent for hydrophilic chemicals, where water’s presence might provide a more suitable extraction environment. Furthermore, the choice of HBDs and HBAs allows adjusting the solvent qualities to fit the physicochemical traits of the bioactive molecules under target control. For some lipophilic compounds, fatty acid-based DESs have been reported to show low viscosity and high extraction efficiency [71]. NADESs made of sugars and organic acids have shown promise in extracting polyphenolic compounds due to their strong hydrogen-bonding capacity [40,72]. García-Roldán et al. demonstrated that betaine/triethylene glycol and choline chloride:1,2-propanediol NADES achieved polyphenol extraction yields comparable to those obtained with 60% ethanol and hot water, while operating at a lower temperature (65 °C) and reduced extraction time. Moreover, the resulting NADES extracts exhibited approximately ten-fold-higher antimicrobial activity than the corresponding aqueous and ethanolic extracts [72]. Cao et al. reported an artemisinin yield of 7.99 ± 0.04 mg g^−1^ from *Artemisia annua* using ultrasound-assisted DES extraction at 45 °C for 70 min, outperforming petroleum ether. The recovery reached 85.65%, and the DES was reusable without notable loss of efficiency [31]. Tsvetov et al. optimized ultrasound-assisted NADES extraction from *Chamaenerion angustifolium* and identified choline-chloride–citric acid as the most effective system, achieving optimal recovery at 58 °C for 35 min with 70 wt% water [73]. Grigorakis et al. reported a maximum total polyphenol yield of 79.93 ± 1.92 mg GAE g^−1^ from *Salvia fruticosa* using a citrate-based DES combined with ultrasonication, outperforming previously developed green extraction approaches [74]. Similarly, Wang et al. achieved a chlorogenic acid yield of 26.39 mg g^−1^ from sunflower seed meal using microwave-assisted ChCl–urea NADES, which also enhanced antioxidant activity compared to ethanol extraction [75]. Moreover, Rodrigues et al. combined COSMO-RS screening with ultrasound-assisted DES extraction and demonstrated efficient recovery of coumarins at 313 K within 30 min, highlighting the role of predictive solvent design in improving extraction selectivity [76].

Moreover, the use of DESs together with contemporary developments in extraction technology, such as MAE and UAE, produced additional bioactive components from bioactive component extraction. UAE mixed with DESs is a strong method that can destroy the structure of the cell wall to release the bioactive compounds from within the cells [77,78]. Likewise, MAE and DESs have been used to get bioactive components, where the recovery rate was substantially greater when compared to conventional methods [79]. However, there are contradicting findings about the efficiency of a given technique when MAE and UAE performance are compared [80]. Overall, these studies show that DES/NADES systems enable efficient extraction of bioactive compounds under mild conditions (typically 30–65 °C and within 60 min), often achieving higher yields than conventional solvents. By adjusting hydrogen bond donors and acceptors and controlling water content, solvent polarity and viscosity can be tailored to improve mass transfer and selectivity. In addition, ultrasound or microwave assistance further shortens extraction time, while COSMO-RS screening helps reduce experimental trial-and-error. Together, these results highlight DES-based extraction as a practical and sustainable alternative, offering advantages in energy efficiency, solvent reuse, and bioactivity preservation.

**Figure 3:**
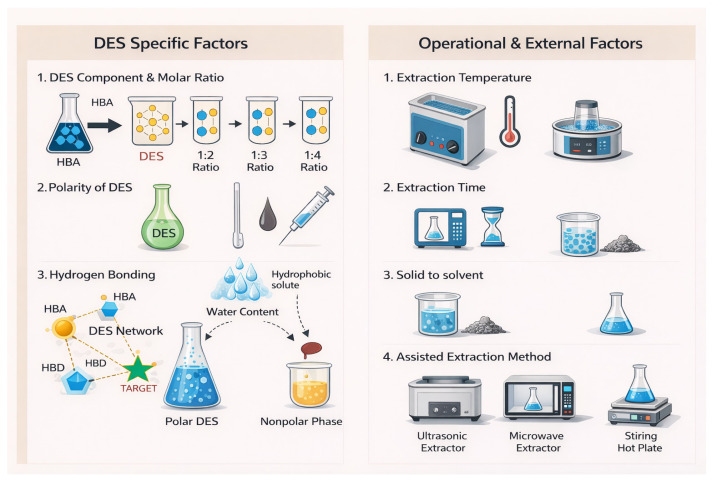
Formation and design principles of deep eutectic solvents and their role in the extraction of bioactive compounds.

Building upon the considerations, this allows the orientation and solvation behavior of the system to be adjusted (Figure 3). The appropriate modification of the HBA/HBD ratio, water content and operating temperature significantly influences extraction efficiency and selectivity. In addition, the viscosity, polarity, and hydrogen-bonding intensity of the DESs directly affect mass transfer, solubility equilibrium, and interactions with target bioactive compounds. The coherent optimization of these parameters enhances the extraction rate and selectivity, thereby determining the overall performance of the process.

## 4. Mechanisms of Extraction with Deep Eutectic Solvents

In this section, the DES-based extraction is interpreted within a process engineering framework. First, the solvent composition (HBA/HBD identity and ratio, water content) determines key physicochemical properties, such as viscosity, polarity, density, conductivity, and surface tension. These properties, in turn, govern mass-transfer phenomena (intraparticle diffusion, film resistance, wetting, mixing, and pumping behavior). Finally, the combination of equilibrium solubility and transport limitations dictates extraction yield, selectivity and process scalability.

### 4.1. Solvation and Physicochemical Interactions

Bioactive compounds are defined as substances that affect living organisms, tissues, or cells and are of great interest because of their biological activity. There is a growing interest in the use of DESs for the extraction and separation of bioactive compounds from complex matrices [81]. This is due to the unique properties and effects of these compounds. This approach is consistent with sustainable practices in chemical processing and improves the efficiency of the extraction process [82].

The dissolution mechanism is naturally influenced by interactions between DES components and bioactive molecules. The main phenomenon in this mechanism is the disruption of the cell wall and the dissolution of target compounds [83]. The key factors affecting the extraction mechanism can be listed as polarity, viscosity, density, conductivity, surface tension and temperature. As a result of the effects of these factors, the interactions between DES components and bioactive molecules are determined.

The type of HBA and HBD and their molar ratio also affect the density, surface tension and viscosity of DESs. Typically, DESs exhibit greater viscosity and reduced conductivity than traditional solvents and ionic liquids. The research indicates that the viscosities of DESs are significantly elevated, with the majority above 100 cP [48,84]. The high viscosity of DESs is attributed to the common hydrogen bonding between the components. This limits the mobility of bioactive molecules in DESs. This is because high viscosity can lead to mass-transfer limitations, which are barriers to the extraction process. Additional interactions, including Van der Waals and electrostatic forces, may also contribute to the elevated viscosity of DESs [85]. These solvent-related factors influence extraction selectivity by regulating the solvent polarity, hydrogen-bonding capacity, and molecular accessibility to specific classes of bioactive compounds. NADESs composed of organic acids and sugars or polyols exhibit enhanced selectivity towards phenolic acids and flavonoids because of the strong hydrogen bonding interactions between the solvent hydroxyl groups and phenolic functional groups. The water content further fine-tunes the selectivity by reducing the viscosity while preserving the hydrogen bonding interactions. Moderate dilution increases mass transfer, whereas excessive dilution weakens the solvent–solute interactions and reduces the selectivity. These examples demonstrate that the rational adjustment of NADES composition enables targeted extraction from natural matrices [80,86].

**Figure 4:**
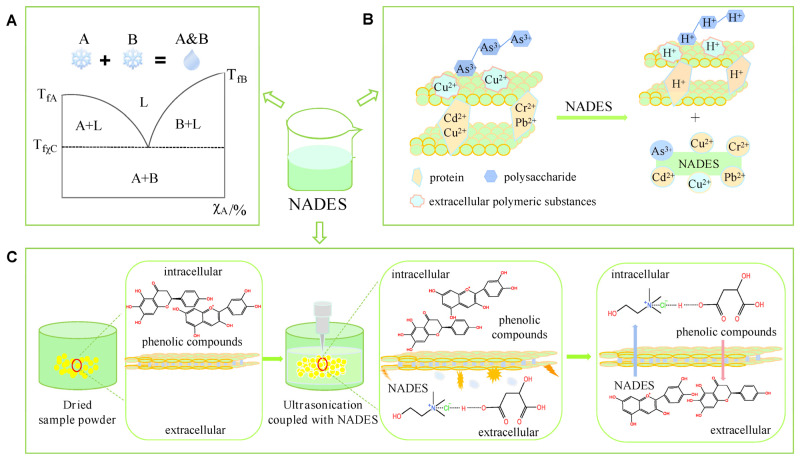
Mechanism of natural deep eutectic solvent (NADES) formation and applications. (**A**) Schematic representation of NADES formation through hydrogen bond interactions between hydrogen bond donors and acceptors; (**B**) illustration of the hydrogen-bonding network responsible for the unique physicochemical properties of NADES; (**C**) schematic representation of the influence of NADES structure on viscosity and freezing point.

In order to perform an effective extraction, DES viscosity can be changed in several ways. The first of these is the addition of water. Adding water to the DES medium increases the mobility of the solvent and facilitates the recovery of bioactive compounds [87]. Nonetheless, certain limits exist. Excessive water addition can weaken the hydrogen bond interactions between DES components, which can lead to undesirable results. As schematically shown in Figure 4, DESs are formed via hydrogen bonds between HBAs and HBDs, creating a dense supermolecular network. This hydrogen bond network is responsible for the inherently high viscosity of DESs. This can limit the molecular mobility and diffusion of target compounds within the solvent matrix. The arrows in Figure 4 represent the hydrogen bond interactions linking the HBA structure to the high-ligand HBD molecules. The interconnected lines show the formation of a continuous network that governs both viscosity and thermal behavior. Additionally, the diagram shows how these strong hydrogen bond interactions contribute to a lower freezing point compared to the individual components, thus stabilizing the solvent system. This structured network enhances the dissolution capacity of DESs for bioactive compounds, while excessive viscosity can hinder extraction efficiency. Therefore, Figure 4 conceptually links hydrogen bond strength, viscosity, and freezing point, highlighting the need for a balanced solvent structure [88]. The hydrogen bond interactions also lower the freezing point of the mixture compared to individual components, further stabilizing the solvent system. Consequently, these properties not only enhance the solubilization of bioactive compounds but also highlight the importance of controlling viscosity and hydrogen bonding for efficient extraction. The tunable nature of NADESs, including adjustment of component ratios and water content, allows optimization for specific extraction tasks.

High viscosity may restrict the diffusion of target compounds into the solvent, whereas low viscosity allows better penetration of the solvent into plant matrices and thus increases the solubility and extraction of bioactive compounds [89,90]. For example, studies have shown that the extraction of flavonoids and phenolic compounds from plant materials is more efficient when DESs with optimized viscosity are used, as this leads to improved solubility and extraction kinetics [85]. While the high viscosity of DESs can contribute to the stabilization of certain solute–solvent interactions, it also imposes significant limitations on mass-transfer rates. This can hinder the diffusion of bioactive compounds, particularly in solid–liquid extraction systems, and may require additional measures such as heating, dilution, or the addition of co-solvents. From an industrial perspective, viscosity directly affects the pumping, mixing, and handling of DESs, thereby influencing scalability and process economics.

Density is one of the most important physicochemical properties of a solvent, along with viscosity and polarity, and significantly affects its solubility. The densities of the most commonly used DESs reported in the literature range from 1.04 to 1.63 g cm^−3^ at 25 °C [91]. The different molecular compositions of DESs are thought to be the main factor determining their density [89]. In most cases, the densities are higher than those of the individual starting materials. This situation is explained by the concept of hole theory. A combination of the DES components results in a reduction in the average radius of the holes, thereby enhancing the density of the DES in comparison to the densities of the individual constituent components [87]. At this point, the molar ratios of the DESs are the main factor determining the density of the DESs. The density of a DES is significantly influenced by the molar ratio of its components. A prior investigation indicated that an elevated ratio of ChCl to glycerol (HBD) led to a reduction in the density of the mixture. It has also been observed that the density of water-retaining DESs changes when exposed to air, and the presence of water also affects the density of DESs [92].

Surface tension is another important physicochemical property for using deep eutectic solvents at the interface and in the colloidal space. However, the amount of research on this topic is rather limited. The surface tension properties of 50 commonly used types of DESs were investigated in the study by Chen et al. [93]. As a result of the studies, both HBD and HBA were found to have a significant effect on the surface tension of DES. It was also observed that the presence of crystal water in the salt component of DESs reduces the surface tension of DESs. Cohesion is the main determinant of fluid surface tension, with stronger intermolecular forces generally resulting in higher surface tension values.

The reduction in surface tension increases the solubility of bioactive compounds and facilitates their extraction from plant materials and other sources, highlighting the significance of this relationship [94]. While early studies established the fundamental relationship between hydrogen bonding and DES viscosity, recent post-2020 investigations have provided molecular-level validation and design-oriented insights, reinforcing the relevance of hydrogen bond engineering in modern DES applications.

Recent studies have further refined the mechanistic understanding of DES viscosity by combining experimental measurements with molecular-level analysis. Ruan et al. demonstrated that the high viscosity of DESs originates from dense hydrogen-bonding networks between HBA and HBD components, which directly affect molecular mobility and mass transfer during extraction. Moreover, the authors highlighted that DES viscosity and selectivity can be systematically tuned by adjusting solvent composition and water content, providing a mechanistic basis for solvent design in separation processes [95].

Mechanistic insights into hydrogen bonding in DES systems have also been supported by recent biomass processing studies. Roy et al. showed that lactic acid-based DESs form strong hydrogen-bond and ion–dipole interactions with biomass components, which govern solvent viscosity and enhance selective solubilization. These findings further confirm that hydrogen bond strength, rather than viscosity alone, plays a key role in controlling DES performance [96].

From an engineering and process design perspective, the effect of the physicochemical properties of DESs on extraction efficiency can be interpreted through mass-transfer mechanisms. High viscosity significantly reduces the effective diffusion coefficient of solutes in the solvent phase and increases internal and external mass-transfer resistance during solid–liquid extraction. Consequently, extraction kinetics in high-viscosity DES systems are generally diffusion-limited.

Increasing temperature and adding water reduces solvent viscosity, increasing molecular mobility and reducing boundary layer resistance at the solid–liquid interface. This explains why most DES-based extraction studies report improved extraction efficiencies at moderately high temperatures and moderate water content. However, excessive dilution disrupts hydrogen bond networks, reducing solvent–solute affinity and selectivity.

These observations suggest that DES extraction performance is governed by a balance between solvent–solute interaction strength and mass-transfer efficiency. From a process design perspective, optimum DES systems should operate within a viscosity range that provides sufficient hydrogen bonding for selective dissolution while maintaining adequate diffusion rates for efficient mass transfer. It is understood that the most important factor affecting the mechanism of action of DESs is their solubility. In addition, the fact that their properties, such as viscosity and density, can be changed significantly affects the extraction processes.

### 4.2. Role of Hydrogen Bonding and Polarity

Beyond general physicochemical parameters such as viscosity, density, and surface tension, solvent polarity and hydrogen bonding play a central role in governing the selectivity and efficiency of DES-based extraction. Extraction performance generally follows the “like dissolves like” principle, whereby solvents with polarity comparable to that of the target compounds exhibit enhanced solubilization and higher extraction yields [97]. The polarity of DESs can have a significant effect on extraction performance. The polarity ranges of deep eutectic solvents vary depending on their composition. The HBD, one of the two main components of DESs, is thought to influence DES polarity. The hydrogen donor and acceptor, interacting via hydrogen bonding, generate a solvent with a lower melting point than when they are alone. Their interactions form an extended hydrogen-bonding network, which not only depresses the melting point but also enables tunable solvent polarity tailored to specific bioactive compounds [98,99]. This tunability is a key advantage of DESs over conventional solvents, allowing them to dissolve a wide range of polar phytochemicals efficiently [100].

Moreover, polarity is affected by temperature through the disruption of hydrogen bond dissociation, while water increases polarity by disrupting intra-DES interactions. Temperature leads to the violation of the hydrogen bond that results in a change in viscosity, polarity and solubility, all of which affect the extraction efficiency [66]. For instance, the melting point of ChCl-urea (1:2) is lower than that of its components (302 °C for ChCl and 133 °C for urea), which shows how molar ratios affect the behavior of DESs. The possibility to control the polarity of the DESs as a function of temperature opens up the opportunity to use them for application in the extraction of polar compounds, separation of biomaterials and solution of poorly soluble substances. Nevertheless, there is a need for further study to enhance their effectiveness for specific tasks [53].

This ability to adjust polarity is one of the main advantages that distinguishes DESs from conventional solvents. It allows researchers to design solvent systems that are specifically suited to different molecular targets instead of relying on a single polarity range. However, the current understanding of how polarity quantitatively correlates with extraction efficiency is still limited. Establishing such correlations would help move DES development from an empirical process toward a more predictive, mechanism-driven design.

Siddiqui et al. stated that it is important to match the polarity of the DESs with that of the target compounds when extraction is performed [101]. Organic acid-based DESs are recommended for the extraction of highly polar phenolic compounds owing to their high polarity. For example, in a study on foxtail millet bran, organic acid-based DESs such as citric acid (Gly-Ca) and betaine (Bet-Gly), with glycerol (Gly) as HBA, had the highest TPC. These findings are significant as they show how the optimization of the DES formulation can be used to enhance hydrogen bonding with polyphenols. Viscosity is another important factor that determines the extraction efficiency, as high viscosity hinders mass transfer and solvent penetration into the plant material, thereby reducing the leaching of bioactive compounds. Zheng et al. observed that Gly-Ca had better extraction efficiency than Bet-Gly even though the latter was slightly more polar. This means that there is a need to find a balance between the viscosity and polarity to achieve the best extraction. Furthermore, surface tension is another factor that determines the capacity of DESs to penetrate plant tissues and affect the extraction yield. Reduced surface tension increases the solubility of phenolic compounds by facilitating cell wall penetration. Hence, for bioactive compound extraction, the design of the DESs should include consideration of important physicochemical properties such as viscosity and surface tension, in addition to polarity [102].

In conclusion, these results demonstrate that no single parameter, including polarity, viscosity, or surface tension, can comprehensively elucidate the efficacy of extraction in isolation. These physicochemical properties, however, interact in subtle ways to determine the behavior of a solvent. Future research ought to focus on experimentally disentangling these effects and developing a unified framework that links molecular interactions within the DESs to the resulting extraction outcomes. This kind of knowledge would help us make a DES formulation that does not need as much trial and error. In summary, solvent polarity should be considered in conjunction with transport phenomena, not as an isolated parameter. While polarity governs solvent–solute affinity and thermodynamic solubility, extraction efficiency is often limited by mass-transfer resistance in high-viscosity DES systems. Increasing polarity through stronger hydrogen bonds can improve solubility but also reduce diffusion rates. Consequently, maximum polarity does not necessarily result in maximum extraction efficiency. Therefore, optimum DES formulations operate within a polarity range that balances increased solubility with acceptable mass transfer and diffusion performance.

**Figure 5:**
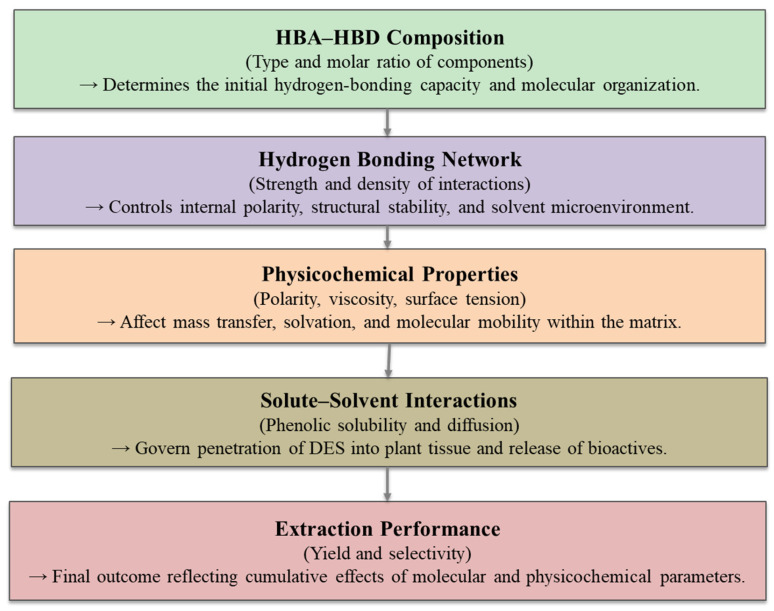
A conceptual flowchart that illustrates the impact of the structural and physicochemical properties of deep eutectic solvents on their extraction efficiency. Arrows indicate the sequential relationship between the stages of the extraction process.

Figure 5 shows that the effectiveness of DES-based extraction comes from a set of parameters that link the molecular structure of the solvent to its performance on a larger scale. This flow clearly shows how the HBA-HBD composition affects the hydrogen-bonding network, which in turn affects the important physical and chemical properties and finally the extraction behavior. In addition to these molecular effects, there are also operational factors that affect how well DES extraction works. These will be discussed in the next section.

### 4.3. Factors Affecting the Extraction Performance

**Figure 6:**
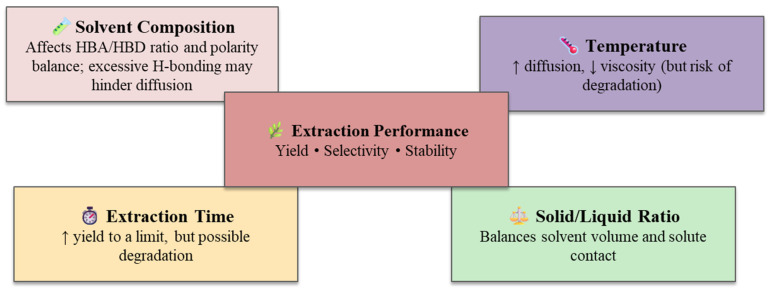
Operational factors influencing DES-based extraction performance. Arrows indicate the influence of the operational parameters on extraction performance.

The main operational conditions that affect the efficiency of the DES-assisted extraction are temperature, extraction time, HBA and HBD molar ratio and solid-to-liquid ratio. These factors affect the solvent penetration, mass-transfer rates and recovery of the bioactive compounds. Figure 6 shows a schematic diagram of these key parameters and how they relate to each other. It shows how each operational factor affects the overall extraction performance of DES-based systems. Solvent composition determines polarity and hydrogen-bond strength, while temperature, extraction time, and solid-to-liquid ratio control viscosity and diffusion, collectively defining yield, selectivity, and stability. The effectiveness of DES-assisted extraction depends on how these factors work together to affect viscosity, polarity, solvation ability, and diffusion behavior. Knowing how they work together makes it possible to design extraction systems that are both high-yielding and long-lasting.

Besides the intrinsic physicochemical properties of deep eutectic solvents, the nature of the solid matrix itself should not be overlooked, as the structural and physical characteristics of the raw material can substantially influence how efficiently bioactive compounds are released into the solvent. In particular, moisture content and particle size play a central role because they directly affect solvent penetration, diffusion pathways within plant tissues, and the overall rate of mass transfer in solid–liquid extraction systems [103,104,105]. In conventional extraction systems, appropriate drying is frequently associated with improved phenolic recovery because reducing the amount of free water minimizes dilution of the extraction medium and limits competition between water molecules and the solvent for interaction sites within the plant matrix. Conversely, excessive residual moisture may weaken solvent–solute interactions and reduce the driving force for diffusion, ultimately lowering extraction efficiency [103,104,105].

Comparable behavior has also been observed in NADES- and DES-based extraction systems. In these media, the amount of water present—either naturally retained in the biomass or deliberately added to adjust solvent properties—can noticeably influence extraction performance. Changes in water content affect viscosity and modify the internal hydrogen-bond network of the solvent, which in turn impacts diffusion and solute mobility [106,107]. Because many DES formulations are inherently viscous, even modest shifts in moisture levels may alter internal transport phenomena and change the resistance to mass transfer within the system [106]. Particle size represents another structural parameter that shapes extraction efficiency. As particle dimensions decrease, surface area increases and the distance that solutes must diffuse inside the matrix becomes shorter. This generally promotes faster solvent penetration and improved release of phenolic compounds, as reported for pear pomace and similar plant residues [105]. Nonetheless, excessive size reduction can create practical limitations. Very fine particles may compact, form dense suspensions, or impede effective mixing, thereby restricting solvent exchange at the solid–liquid interface. Consequently, moisture content and particle size should be considered alongside DES composition and operating variables. Optimizing these factors together, rather than in isolation, supports the development of extraction processes that are not only efficient but also scalable and economically viable [107].

From a process-engineering perspective, these operational parameters should not be viewed merely as adjustable laboratory variables. In real processing environments, extraction time, solvent-to-feed ratio, and HBA/HBD composition interact with each other and directly affect mixing efficiency, mass-transfer behavior, and solvent management. A process that appears efficient under laboratory conditions does not always maintain the same behavior once larger processing volumes are introduced. High viscosity, for example, can limit agitation efficiency and create pumping challenges. Likewise, increasing the solvent-to-feed ratio may enhance extraction yield, but it also raises solvent consumption, recovery requirements, and overall energy demand. For this reason, optimization should extend beyond maximizing extraction performance alone. Practical feasibility, solvent recyclability, equipment compatibility, and operational stability under continuous or semi-continuous conditions must also be considered. Considering these aspects together is essential if DES-based extraction is to progress beyond laboratory demonstrations and become a technically and economically feasible large-scale process.

#### 4.3.1. Effect of Temperature on the Extraction Performance

Extraction temperature is a crucial parameter for improving the bioactivity and extraction yield of phenolic compounds. Normally, the density, viscosity and surface tension of DESs decrease with an increase in extraction temperature, thus facilitating the penetration of biomass cells and increasing the interactions between DESs and target compounds. At high temperatures, the energy that is accumulated by the DES molecules makes it possible for the molecules to move freely. This process breaks hydrogen bonding and van der Waals forces and leads to viscous flow that enhances the extraction of phenolic compounds. Also, high temperatures induce structural changes in the DESs through the rearrangement of anions and cations, which lowers the viscosity and increases the conductivity. The solubility and diffusion of the phenolics from the solid phase to the liquid phase are greatly enhanced at higher temperatures because of the increased interactions in the solution. This is because phenolic compounds are usually bound in the solid phase by hydrogen bonding, van der Waals forces, and electrostatic interactions; thus, increasing temperature weakens these forces and facilitates their dissolution in the DESs [108]. However, there is a disadvantage of the high temperatures, which includes the possibility of degrading some compounds. It is therefore very important to optimize the temperature for the purpose of obtaining maximum phenolic extraction with minimal phenolic degradation [101]. Achkar et al. also pointed out that those changes in the hydrogen bond network with temperature can alter the solvent properties. Therefore, precise temperature control is required for the best extraction [66]. The optimal extraction temperatures of different plant matrices in different plant sources could be attributed to the sensitivity of the target compounds and the physical characteristics of the solvent system.

When soy-based products were extracted with DESs, compounds such as daidzin, daidzein, genistin and genistein were found to have improved solubility in the DESs with increasing extraction temperature from 10 to 60 °C [109]. Extraction yield did not increase further with temperature above this point, indicating that a plateau had been reached where temperature added nothing to the rate of compound diffusion. Likewise, the flavonoid recovery was highest in pigeon pea leaves at 60 °C, specifically for orientin and luteolin. However, when the temperature was above 70 °C, some of the target compounds were either unaltered or the extraction yield was reduced owing to the degradation [110]. Some polyphenols possess high thermal stability and can be extracted efficiently at high temperatures. The highest extraction yields of catechin from red grape skin were obtained at 80 °C, while quercetin-3-O-glycoside started to decompose at 65 °C [111]. The optimum recovery of gingerol from ginger was at 50 °C, while the compound was greatly degraded above 60 °C [112]. However, there are some DES systems that are effective over a wide range of temperatures. When ChCl-ethylene glycol was used, the extraction yield was enhanced with increasing temperature from 0 to 70 °C for catechin and epigallocatechin gallate from Chinese green tea. However, the extraction yield did not change much when the temperature was raised from 95 °C, indicating that there is a limit beyond which thermal effects are not the main determinant of the extraction yield [113]. These observations suggest that temperature needs to be well controlled during the DES extraction process. While moderate heating normally improves the diffusion and solubility of the extract, high temperatures may degrade the phenolic compounds and lead to a reduction in the extract yield. Therefore, choosing an appropriate temperature is crucial for obtaining the maximum yield of polyphenols with optimal activity. In summary, temperature not only regulates mass transfer but also defines the thermal stability range of bioactives. Thus, identifying a balanced thermal regime—high enough to decrease viscosity yet low enough to prevent degradation—is key to sustainable and reproducible DES-based extraction.

From a process-engineering standpoint, temperature plays a central role in balancing mass transfer and product stability in DES-based extraction. Taken together, the reported studies indicate that DES-based extraction of phenolics and flavonoids is typically carried out within a moderate temperature window of approximately 30–65 °C. Within this range, the temperature is high enough to reduce viscosity and improve diffusion, yet still mild enough to avoid marked thermal degradation of heat-sensitive bioactive compounds. At lower temperatures, the higher viscosity of many DESs makes mass transfer slower, whereas very high temperatures tend to damage sensitive compounds and add unnecessary energy costs.

#### 4.3.2. Effect of Extraction Time on the Extraction Performance

In this subsection, the reported extraction times refer to dynamic DES-based solid–liquid extraction protocols, in which the biomass is contacted with the solvent under continuous stirring or ultrasound assistance rather than static maceration [100,101,108,109,110,111,112,113,114,115,116,117]. In many DES applications for the recovery of phenolic compounds from soy products, grape skins, Cajanus cajan leaves, ginger, foxtail millet bran, mangosteen peel, *Juglans regia* and *Morus alba*, ultrasonic-assisted or mechanically agitated batch extraction is employed, where hydrodynamic renewal of the solid–liquid interface and, in the case of UAE, acoustic cavitation accelerate mass transfer and allow equilibrium to be reached within comparatively short contact times [101,108,109,110,111,112,113,114,116,117]. Under comparable conditions, purely static soaking with DESs would typically require longer extraction times to achieve similar yields because mass transfer is controlled mainly by molecular diffusion in a nearly stagnant boundary layer [100,101,102,115].

The extraction time is a fundamental parameter that determines the mass-transfer kinetics in the solid–liquid system since it plays an important role in determining the yield and quality of extracted bioactive compounds. As the process progresses, the diffusion rate, which is initially high, gradually decreases. Then, the extraction kinetics become stationary when the equilibrium between the solid and liquid phases is achieved. Initially, an optimal extraction time enhances the recovery of phenolic compounds. However, prolonged extraction may lead to oxidation, hydrolysis, or polymerization, all of which negatively affect bioactivity [108]. Therefore, it is necessary to optimize the extraction time to obtain the maximum yield with minimal alteration in the structure of the extracted compounds. The extraction yield is mainly dependent on the plant material and the solvent used. For example, in *Setaria italica*, the optimum polyphenol extraction was achieved with betaine/glycerol (1:5) at 30 min, even though the yield was somewhat lower at 50 min due to thermal degradation [102]. In contrast, in *Garcinia mangostana* L., the best result was obtained with choline-chloride/lactic acid (1:2) at 15 min, while a much lower yield was observed at 1 min, which may be because of the fact that phenolics remained bound in the plant matrix and did not diffuse fully into the solvent within such a short time [116]. Some plant matrices are structurally complex and may need to be sonicated for longer times. For instance, in *Juglans regia* L., the highest extraction yield was achieved with ChCl:phenylpropionic (1:2) at 105 min, which indicates that perhaps longer extraction times might be required for tough cell walls [117]. However, the optimum extraction yield was obtained at 30 min for *Morus alba* L. [114]. However, when extraction was carried out for 50 min, a substantial degradation was observed. The extraction time of phenolic compounds varies according to the solvent system and plant material, and usually ranges between 30 and 240 min [108]. Nevertheless, exceeding the optimal time can lead to solvent degradation and a change in the physical properties of DESs. From an industrial point of view, the additional extraction time may not lead to a significant increase in the yield but will lead to an increase in energy consumption and compound degradation. Alam et al. pointed out that the prolonged extraction time does not necessarily lead to improved effectiveness and may even result in reduced bioactivity of the extract because of the oxidation and degradation of active compounds caused by exposure to light and air [108]. Therefore, it is important to consider the extraction yield and compound stability to obtain maximum recovery of the compounds with maintained functional properties. In conclusion, the extraction time represents a kinetic control parameter that defines the balance between solute diffusion and degradation. Identifying the shortest effective duration improves extraction efficiency and enhances process reproducibility and compound stability in DES-based systems.

From a process design standpoint, extraction time is closely linked to the solvent-to-feed (solid–liquid) ratio, since both parameters jointly determine the driving force for mass transfer and the approach to equilibrium. Similar interdependencies between contact time and solid–liquid ratio have been highlighted in recent DES-based extraction studies (e.g., Vieira et al. for *Juglans regia* leaves and Zheng et al. for foxtail millet bran), where increasing the solvent-to-feed ratio allowed shorter extraction times to reach comparable phenolic yields, while excessive solvent usage only marginally improved recovery but increased downstream costs [102,117]. At higher solvent-to-feed ratios, the concentration gradient between solid and liquid phases is larger, and saturation of the solvent occurs later, so comparable phenolic yields can often be achieved within shorter extraction times; conversely, when the solvent loading is reduced, longer contact times are usually required to reach similar recoveries. However, excessively high solvent-to-feed ratios combined with long extraction times only marginally improve yield while increasing solvent consumption and downstream concentration and recycling costs, which is why DES-based extractions are typically optimized by simultaneously adjusting extraction time and solid–liquid ratio to identify the shortest effective contact time at an intermediate, economically viable solvent usage.

From a process-engineering perspective, extraction time should not be treated as an independent variable, since it is closely linked to the solvent-to-feed ratio, hydrodynamics and solvent viscosity. In well-agitated or ultrasound-assisted laboratory systems, these conditions often make it possible to reach near-equilibrium yields within relatively short times. However, in large-scale operations, longer diffusion distances and lower mixing efficiencies can considerably increase the time required to obtain similar recoveries. Therefore, choosing an appropriate extraction time requires weighing the kinetic advantage of extended contact against the additional energy demand and the risk of degrading heat- or oxidation-sensitive bioactives, especially when scale-up is involved.

#### 4.3.3. Effect of HBA:HBD Molar Ratio on the Extraction Performance

Several studies have established that the HBA:HBD ratio is a critical determinant factor that enhances the yield of phenolic compounds during extraction. However, a certain imbalance may lead to undesirable viscosity effects or weakened hydrogen bonding interactions, which is not desirable for extraction efficiency. This section will discuss the effects of the variations in the HBA:HBD ratios on the physicochemical properties of the DESs and their efficiency in extracting phenolic compounds from plant materials.

Recent studies have shown that the glycerol fraction in glycerol-based DESs for orange peel extraction decreased the efficiency of extraction because of the increased viscosity that retarded mass transfer. Conversely, increasing the fraction of ethylene glycol from 1:2 to 1:4 improved extraction efficiency by reducing viscosity and enhancing solvation properties [118]. Furthermore, Abbasi et al. observed that in choline-chloride-based polyalcohol DES systems, the HBA:HBD ratio is an important factor that regulates the viscosity and mass transfer as well as solvation and extraction efficiency [115]. They reported that the hydrogen bond basicity was constant at a certain level, whereas hydrogen bond acidity was at its maximum in the systems with a 1:10 HBA:HBD ratio. However, the high HBD content caused increased viscosity that lowered the extraction yield. A similar trend was observed in *Pyrola incarnata* Fisch., where the optimal ratio for polyol-based DESs was determined to be 1:4 ChCl to polyol. Exceeding this ratio led to decreased efficiency because of weakened chloride anion interactions [119]. Similarly, in *Flos Sophorae Immaturus*, rutin, quercetin, and kaempferol extraction peaked at a 1:2 ChCl to 1,4-butanediol ratio, but further dilution to 1:5 resulted in reduced extraction efficiency [120]. In the extraction of *Equisetum palustre* L., the ChCl:ethylene glycol ratio was reduced from 1:1 to 1:2, which increased the extraction yield. Further reduction to 1:4 was adverse owing to the suppressed activity of ChCl [121]. Öztürk et al. reported that the HBA:HBD molar ratio significantly influenced polyphenol extraction from orange peel. Under base conditions (30 wt.% water, 313.15 K), TPC increased from 1.37 mg GAE/g OP for [Ch]Cl:Gly 1:4 to 2.90 mg GAE/g OP for [Ch]Cl:Gly 1:2, corresponding to a nearly 2.1-fold enhancement when the HBA proportion increased. Similarly, for ethylene glycol-based DES, TPC varied from 1.29 mg GAE/gOP ([Ch]Cl:EG 1:2) to 2.27 mg GAE/g OP ([Ch]Cl:EG 1:4), indicating 76% variation depending on molar ratio [118]. Cheng et al. screened 6429 HBA-HBD combinations in four molar ratios (2:1, 1:1, 1:2, 1:3) for α-tocopherol extraction. Among them, 394 DES candidates ranked in the top 10% for both infinite dilution capacity (C^∞^ > 3.11) and selectivity (S^∞^ > 8.08) [122]. Cao et al. showed that lowering the molar ratio of HBD to HBA enhanced the extraction efficiency of artemisinin [31]. Maaiden et al. confirmed that hydrogen bonding strength varies directly with molar ratio, affecting mass-transfer efficiency [123]. De Faria et al. also demonstrated that increasing the alkyl chain length of DES components enhanced cynaropicrin yield by up to 18% [124]. Hence, it is clear that the chemical nature and molar ratio of these components are absolutely essential for optimizing extraction processes. In essence, the molar ratio of HBA to HBD is a basic determinant of the bioactive component extraction efficiency. Changing this ratio maximizes the physical characteristics of the DES, improving the extraction mechanism for several bioactive substances, as shown by several studies in different uses [8,125].

Besides viscosity and solubility, the density of the DESs is also influenced by the HBA:HBD molar ratio. It has been reported that the density increases with the use of certain HBDs, such as citric acid or polyethylene glycol, since this may affect mass transfer and solvation efficiency. Therefore, the HBA:HBD ratio should be optimized not only to control the viscosity but also to achieve the best extraction conditions [126]. Moreover, the research has revealed that the excessive dilution of ChCl with alcohol-based HBDs weakens hydrogen bonding and thus the extraction yield [127]. Increasing the glycerol content (2:1 to 1:2 molar ratio) in the ChCl-glycerol-based DESs enhanced the yield of phenolic compounds through improved diffusion and mass transfer during the extraction of neochlorogenic acid, chlorogenic acid, cryptochlorogenic acid, caffeic acid, rutin, isoquercetin, and astragalin from mulberry leaves [128]. However, increasing the glycerol content to 1:5 resulted in reduced efficiency because of the increased steric hindrance and the weakened chloride anion interactions with the target compounds. Overall, HBA:HBD molar ratio is a key determinant of the physicochemical properties of such systems. While increasing the amount of HBD component can improve solvation and hydrogen bonding, a large amount of HBD brings high viscosity and low mass-transfer efficiency. In conclusion, the HBA:HBD molar ratio defines the physicochemical landscape of DESs by shaping their viscosity, polarity, and hydrogen-bond network. Identifying the composition that ensures optimal solvation and diffusion without excessive viscosity is critical for achieving efficient, reproducible, and environmentally sustainable extraction of bioactive compounds.

From a process-engineering standpoint, the HBA:HBD molar ratio plays a central role because it directly affects hydrogen-bond interactions, solvent polarity and viscosity. Increasing the HBD fraction often improves the solvation of phenolic and flavonoid compounds, but it also tends to raise viscosity and slow down mass transfer. In contrast, HBA-rich mixtures usually exhibit lower viscosity, although specific solvent–solute interactions and selectivity may be weakened. In many reported DES systems, intermediate HBA:HBD ratios provide a practical compromise, maintaining sufficient solvation strength while avoiding excessive viscosity, and are therefore more suitable when extraction efficiency, solvent handling and scale-up are considered together.

#### 4.3.4. Effect of Solid/Liquid Ratio on the Extraction Performance

The appropriate solid/liquid ratio minimizes saturation and maximizes solubility of bioactive compounds, allowing the solvent to penetrate the cell matrix rather than dispersing into the bulk phase. Hence, too low a ratio leads to poor extraction, whereas too-high solvent loading represents a waste in terms of economy as well as being environmentally unfriendly.

Ozbek Yazici and Ozmen (2022) reported that a solid/liquid ratio of 1:30 achieved the highest yield for the fruit of *Capparis ovate* [129]. There was no further increase in extraction efficiency beyond this level [129]. Another study claimed that the highest yield was reached at a solid/liquid ratio of 1:30 for the apple pomace extraction, after which the yield would drop due to the further dilution of the solvent [130]. As a result, increasing the amount of solvent allows the extraction efficiency to be enhanced at certain levels. Additionally, it has been shown that further addition of solvent does not enhance efficiency beyond a certain saturation point.

From a transport perspective, the solid-to-liquid ratio governs the concentration gradient between the biomass and the solvent phase. At low solvent volumes, rapid saturation of the DES reduces the driving force for diffusion and limits extraction yield, whereas increasing solvent volume helps to maintain this gradient for longer and thus enhances mass transfer. However, excessive solvent loading increases downstream solvent recovery requirements, mixing demand and overall energy consumption. Therefore, optimal solid-to-liquid ratios should balance the mass-transfer driving force with solvent management and recovery feasibility, particularly when scaling up DES-based extraction systems.

In practical operation, the solid-to-liquid ratio determines how much solvent is required and how demanding the recovery step will be. While sufficient solvent volume is needed to sustain effective diffusion, excessive solvent use increases handling complexity and energy demand during downstream recovery. As illustrated in Figure 6, the solid-to-liquid ratio interacts with solvent composition, temperature and extraction time through their combined effects on viscosity, diffusion behavior and solute accessibility. Considering these parameters together provides a clearer basis for developing DES-based extraction systems that remain efficient at a larger scale.

The choice of HBA and HBD components, their proportions and the amount of water added strongly influence basic solvent properties such as viscosity and polarity. These, in turn, affect how easily molecules move in the liquid and how well mixing and mass transfer take place, so extraction yield and selectivity depend on both solvent–solute interactions and how efficiently species are transported through the medium. Considering these factors together, rather than treating them separately, offers a more practical basis for process development and makes it easier to translate laboratory observations into larger-scale applications.

## 5. Cost-Effectiveness and Environmental Impact

DESs present several layers of economic and environmental advantages that go beyond their smaller required quantities. The ingredients of this solvent system generally involve inexpensive, abundant and often natural components (typically food-grade and biodegradable), as already described above [45,46]. Additionally, the production process is simple and energy-efficient. Unlike conventional organic solvents that necessitate multi-step distillation and solvent recovery, this process requires only mild heating. Furthermore, there is no need for a purification step. Thus, this leads to reducing energy and production costs.

On the other hand, although the initial cost of individual DES components might vary, their recyclability and reusability markedly improve cost-efficiency. Multiple studies have reported that DESs can be reused for several extraction cycles (up to 3–5 times) with minimal loss of extraction efficiency, leading to a lower cost per extraction and reducing solvent waste [131,132,133]. For instance, Jeong et al. demonstrated the successful tailoring and recycling of choline-chloride-based DESs for the extraction of bioactive compounds, maintaining high extraction performance over multiple reuse cycles [131]. Similarly, He et al. reported that NADES systems employed for the ultrasonic-assisted extraction of bioactive compounds from Salvia miltiorrhiza retained their efficiency after repeated use [131]. From a broader process-development perspective, DES recovery and recycling strategies have been systematically reviewed and identified as key enablers for scalable and economically viable biomass conversion processes [132]. Such solvent reusability is a critical factor for process scalability, as it directly impacts operating costs, solvent-to-feed ratio optimization, and waste minimization, thereby supporting the industrial feasibility of DES-based extraction systems. In addition, their low vapor pressure minimizes solvent loss during processing, reducing the need for energy-intensive evaporation. This also further decreases the operational costs [23].

From an environmental perspective, DESs produce significantly less volatile organic compound emissions and exhibit low toxicity and high biodegradability, minimizing waste treatment requirements [134]. The combination of cheap raw materials, low energy input, high recyclability, and safer disposal underpins the cost-effectiveness and sustainability of DESs in both laboratory and industrial applications.

## 6. Challenges and Limitations

Regulatory considerations and scalability are crucial for the large-scale commercialization of DES extraction processes. To date, DESs have been primarily explored at pilot and pre-industrial scales in sectors such as nutraceuticals, functional food ingredients, cosmetics and pharmaceutical raw materials. Representative examples include the extraction of polyphenols from grape pomace and olive leaves for nutraceutical formulations, recovery of flavonoids and phenolic acids for cosmetic actives, and isolation of alkaloids and terpenoids as pharmaceutical intermediates. In the food sector, DES-based extraction has been investigated for obtaining natural antioxidants, colorants, and flavor compounds from agricultural by-products, aligning with circular bioeconomy strategies. Despite these promising application areas, widespread industrial adoption remains limited due to regulatory constraints, solvent recovery challenges and compatibility with existing downstream purification systems. In several pilot-scale studies, DES-based extraction of polyphenols from agro-industrial residues such as grape pomace [135] and olive leaves [25] has demonstrated extraction yields comparable to or higher than those obtained with conventional organic solvents, while operating at moderate temperatures and reduced solvent losses. Reported solvent recovery efficiencies after recycling steps typically remain above 80–90% depending on DES composition and separation strategy [133]. Techno-economic and life-cycle assessments available in the literature indicate that, although DES-based processes may involve higher initial energy demand due to viscosity-related mixing and pumping requirements, overall process sustainability can be improved through solvent recycling, reduced solvent toxicity, and integration with intensified extraction techniques. From an equipment perspective, pilot-scale implementations highlight the need for adapted mixing systems, positive-displacement pumps, and temperature-controlled units to handle the high viscosity of DESs and maintain homogeneous solvent composition during continuous or semi-continuous operation [23].

The development of multiple DES formulations into new products has resulted in insufficient research on their safety capacities and permanent effects, with the FDA and EFSA requiring detailed safety checks before allowing their use in pharmaceutics cosmetics and food items [136]. Insufficient criteria for assessing DES toxicity levels and residual amounts within end products prevent regulatory agencies from approving product applications [27]. Selective precipitation with DES frameworks represents an effective strategy for resolving this issue. The method for compound extraction needs specific optimization for individual substances, making it unsuitable for universal application. Researchers have proposed enzymatic degradation and biocompatible co-solvent systems for DES elimination, yet their efficiency depends on the chosen extraction method [137]. The continued presence of DESs in bioactive compound extraction, but their efficiency depends on the chosen extraction method. DESs’ presence in food and pharmaceutical applications poses issues due to strict regulatory standards. The adoption of DES as an extravagant bioactive compound extraction requires confirmation of compatibility with existing downstream purification systems.

The lack of regulatory understanding regarding DES extractions has hindered their large-scale commercial use. The emergence of various DES formulations has led to insufficient toxicity research about their safety performance and persistent effects [138]. Regulatory institutions like the FDA and EFSA require extensive safety assessments before DES applications are approved in pharmaceuticals, cosmetics and food products. The absence of clear procedures for assessing the toxicity level and residual amounts of DES end products hinders regulatory agencies from approving product applications. The regulatory review focuses on the composition of DES materials, favoring naturally sourced food-grade elements in DES production. This makes synthetic quaternary ammonium salt-containing or industrial chemical DES formulations harder to get approved. Although DESs are often described as “green” and “biodegradable,” their toxicity and ecotoxicity profiles strongly depend on their specific composition. Several experimental studies have shown that choline-chloride-based DESs may exhibit low to moderate cytotoxicity depending on the HBD, concentration and exposure conditions. Ecotoxicity assessments using aquatic organisms and cell-based assays have reported variable responses, highlighting the need for compound-specific safety evaluation rather than generalized sustainability claims. Therefore, the classification of DESs as environmentally benign should be made cautiously and supported by systematic toxicological assessment [139,140].

One of the major challenges limiting the practical and industrial implementation of DES-based extraction is the removal or recovery of DESs to obtain solvent-free bioactive extracts. Due to their negligible vapor pressure, DESs cannot be removed by conventional evaporation, making downstream separation more complex than for volatile organic solvents. Strategies such as anti-solvent precipitation, membrane separation, adsorption, back-extraction and enzymatic degradation have been proposed. However, their effectiveness strongly depends on DES composition, target compounds and process conditions. Therefore, efficient DES recovery is essential for regulatory compliance, product purity and whole process sustainability [133,141]. Moreover, variations in water content can significantly alter hydrogen-bonding networks, viscosity, polarity and extraction selectivity, which might lead to reproducibility challenges and reduced process robustness under industrial conditions [26,27].

Another critical limitation concerns the high solvent-to-feed ratios frequently required in DES-based extraction of bioactive compounds from natural matrices. While elevated solvent ratios might enhance solute solubilization and mass transfer, they significantly increase solvent consumption, energy demand, and downstream processing costs, especially at larger scales. This challenge directly affects equipment sizing, solvent recovery efficiency and process economics. Approaches such as process intensification techniques, such as UAE and MAE, DES recycling, and optimization of particle size and moisture content have been proposed to reduce solvent demand and improve industrial feasibility [132].

Research is underway to develop non-toxic and biodegradable DES formulations, but regulatory approval is a challenge due to the lack of specific guidelines. Moreover, industrial DES applications face scaling operations, high costs, and scalability issues due to specialized high-viscosity solvent handling equipment and maintaining uniform DES composition. DES-based processes require equipment modifications to suit their physical characteristics, increasing operational expenses [132].

Further optimization is needed to fully integrate DESs into current industrial production systems. Researchers are also working on creating affordable DES solutions from low-cost raw materials to increase market potential. Despite current challenges, DESs’ adjustable behavior, an infinite range of bioactive molecule extraction, and environmental advantages make them superior alternatives to traditional solvents. To succeed in industrial applications, DESs must address viscosity issues, recyclability and downstream process compatibility, regulatory standards, and scalability limitations. DESs’ establishment as a sustainable bioactive compound extraction solvent relies on continuous research partnerships between academia, industry, and regulatory authorities.

## 7. Future Trends and Perspectives

The future of extraction technologies holds immense promise, offering opportunities to enhance efficiency, sustainability and precision. As these technologies evolve, the demand for more customized and eco-friendly solutions continues to grow. Several emerging trends are expected to shape the next phase of extraction methods, particularly in developing tailored solvents and integrating advanced techniques. These trends will have significant applications in key industries such as pharmaceuticals and nutraceuticals.

Traditional extraction processes often depend on organic solvents, which pose significant risks to both human health and the environment. These methods are typically energy-intensive, time-consuming, and inefficient for certain types of compounds. As a result, there is increasing interest in more sustainable and effective extraction techniques. Recent advances such as UAE, MAE and Dispersive Liquid–Liquid Microextraction (DLLME) have emerged as promising alternatives, enhancing extraction performance while reducing solvent usage. By combining these advanced techniques with DESs, researchers have achieved notable improvements in extraction efficiency and selectivity. UAE utilizes high-frequency sound waves to create cavitation bubbles in the solvent. When these bubbles collapse, they generate tremendous force, leading to localized high-pressure and high-temperature zones. This process disrupts cell walls, facilitating the release of compounds into the solvent and significantly enhancing extraction efficiency [142]. Future research should shift from qualitative sustainability claims to a quantitative and mechanistic understanding of DESs. Key physicochemical variables such as viscosity and polarity govern solute transport and extraction performance. The high viscosity characteristic of many NADESs can impede mass transfer and reduce extraction yields. This highlights the necessity of quantifying these relationships instead of merely describing them qualitatively [48]. Complementary transport measurements show that diffusivities in deep eutectic media depend on composition and operating conditions, reinforcing the value of explicitly relating viscosity and polarity to measurable transport metrics [49]. Recent advances in data-driven modeling and machine learning approaches enable predictive screening of DES compositions and estimation of key physicochemical properties such as viscosity, density, and polarity, thereby reducing empirical solvent screening efforts [143,144]. Data-driven models and machine learning approaches have recently been applied to predict physicochemical properties of DESs, reducing empirical solvent screening efforts [145,146]. At the molecular scale, simulation and electronic-structure tools are already being applied to DES. Quantum-chemical calculations clarify hydrogen-bonding patterns and solvation tendencies, while molecular dynamics simulations (particularly those using machine-learned interatomic potentials) investigate structure–dynamics relationships. These integrated experimental and computational methods offer a reliable approach to minimize trial-and-error in solvent selection and to systematically design next-generation DESs with customized hydrogen-bond networks.

Another crucial aspect is regulatory acceptance and safety profiling. The absence of standardized toxicological data and well-defined limits for residual solvents obstructs the adoption of DESs in pharmaceuticals and food industries [27]. Some reviews note that the application of DESs in the food industry is regulated by organizations such as EFSA [147]. Future studies must define no-observed-adverse-effect-level (NOAEL) thresholds, long-term cytotoxicity assays, and biodegradability protocols such as OECD ready biodegradability tests [27]. Building collaborative frameworks with regulatory agencies (such as EFSA and FDA) could accelerate approval by developing GRAS-compliant solvent libraries and harmonized risk assessment guidelines.

Industrial scalability and economic feasibility also require attention. Pilot-scale continuous systems with closed-loop DES regeneration must be developed and benchmarked. However, there is currently a lack of literature reporting over 80% solvent recovery or over 90% extraction yield under conditions that mimic industrial practices. Life-cycle analysis (LCA) and techno-economic assessment (TEA) are increasingly applied to DES-based biomass processing routes to provide data-driven evidence of real cost and carbon footprint advantages compared to conventional solvents [148]. These methods may guide the translation of DESs from lab to plant.

**Figure 7:**
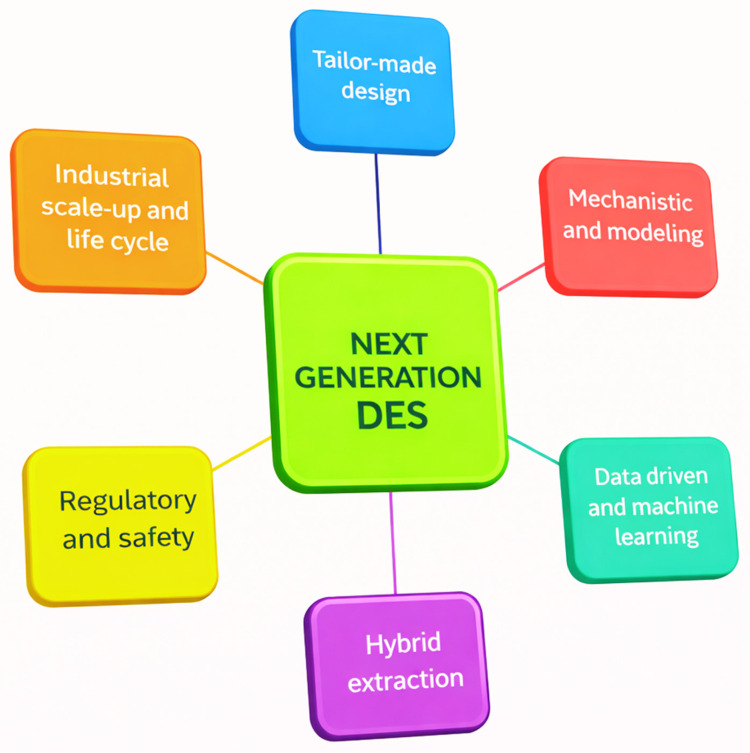
Future perspectives and emerging directions in next-generation DESs.

These future directions emphasize not only the versatility and sustainability potential of DESs but also outline a practical roadmap for their broader adoption (Figure 7). Figure 7 summarizes the key technical and regulatory milestones required for scaling DES-based extraction from laboratory to pilot and industrial operation, including solvent recovery efficiency, solvent-to-feed ratio optimization and equipment adaptation for high-viscosity systems. They integrate concepts of tailor-made solvent design, quantitative and mechanistic understanding of key physicochemical parameters, and the integration of advanced extraction technologies such as UAE, MAE and DLLME. In addition, the need for robust safety profiling and harmonized regulatory acceptance frameworks (such as NOAEL thresholds, OECD biodegradability protocols and GRAS strategies) and industrial scalability supported by TEA and LCA is underlined. Finally, emerging data-driven and machine learning tools offer a pathway to accelerate rational DES design and reduce empirical trial-and-error, supporting the transition from conceptual “green solvents” to safe, efficient, and approval-ready industrial solutions.

## Figures and Tables

**Table 1:** Representative examples of DES systems: structural DES Types (I–V) and compositional/functional variants.

DES System/Variant	HBA	HBD	Main Properties of the DES Type	Reference
Chlorine-based DES	ChCl	Glycerol, urea, ethylene glycol, oxalic acid, citric acid, malonate, levulinic acid, acetamide, sucrose, phenol	Low-cost, biodegradable, low toxicity, widely used in extraction and catalysis; physicochemical properties (viscosity, polarity, acidity) tunable by HBD selection.	[29]
Betaine-based DES	Betaine	Levulinic acid, malic acid, citric acid, lactic acid, ethylene glycol maltose, glucose, xylitol, sorbitol, urea	Bio-based and highly water-soluble DESs; often classified as NADESs; effective for green extraction of phenolics, sugars, and bioactive compounds.	[29,30]
Quaternary ammonium-based DES	Tetrabutylammonium bromide, tetrabutylammonium chloride, tetraethylammonium chloride, tetraethylammonium bromide, tetramethylammonium chloride, tetrabutylphosphonium bromide, ethylammonium chloride, tetrapropylammonium bromide, methyl triphenyl phosphonium bromide	Glycerol, urea, ethylene glycol, triethylene glycol, malonic acid, levulinic acid	Broader range of HBAs compared to ChCl; form liquids near room temperature; suitable for electrochemistry, separations, and catalysis.	[31,32,33,34,35,36]
Metal salt-based DES	Metal chlorides (AlCl_3_, ZnCl_2_, FeCl_3_, SnCl_2_, CuCl_2_)	Glucose, fructose, urea, glycerol, tetrabutylammonium bromide, acetamide and polybasic alcohol	Lewis-acidic DES systems; properties strongly depend on metal salt identity and molar ratio; widely used in catalysis, metal processing, and biomass conversion.	[37,38,39]
Sugar-based NADES	Natural sugars (dual HBA/HBD role)	Glucose, fructose, sucrose ± water/organic acids	Highly biocompatible solvents; suitable for extraction and stabilization of sensitive phytochemicals.	[40]
Organic acid-based NADES	Organic acids	Lactic acid, malic acid, citric acid mixtures	Acidic NADES; excellent solubilization of alkaloids and phenolic compounds.	[41]
Amino acid-based NADES	Amino acids	Proline, glycine + organic acids/polyols	Strong hydrogen bonding; efficient for extraction of flavonoids and antioxidants.	[42]
Hydrophobic NADES	Terpenes (menthol, thymol)	Fatty acids, cineol, natural organic acids	Water-immiscible NADES; selective extraction of nonpolar bioactives (terpenoids, essential oils).	[43]
Ternary NADES systems	Mixed natural metabolites	Sugar + acid + polyol (three-component NADES)	Enhanced tunability of polarity and viscosity; often improves extraction yield compared to binary NADES.	[44]

## Data Availability

The datasets generated during and/or analyzed during the current study are available from the corresponding author on reasonable request.
